# Wheat as a Storehouse of Natural Antimicrobial Compounds

**DOI:** 10.3390/molecules30244774

**Published:** 2025-12-14

**Authors:** Eva Scarcelli, Domenico Iacopetta, Jessica Ceramella, Daniela Bonofiglio, Alessia Catalano, Giovanna Basile, Francesca Aiello, Maria Stefania Sinicropi

**Affiliations:** 1 Department of Pharmacy, Health and Nutritional Sciences, University of Calabria, Via Pietro Bucci, 87036 Arcavacata Di Rende, Italy; eva.scarcelli@unical.it (E.S.); jessica.ceramella@unical.it (J.C.); daniela.bonofiglio@unical.it (D.B.); giovanna.basile@unical.it (G.B.); s.sinicropi@unical.it (M.S.S.); 2 Centro Sanitario, University of Calabria, 87036 Arcavacata Di Rende, Italy; 3 Department of Pharmacy-Drug Sciences, University of Bari “Aldo Moro”, Via Orabona, 4, 70126 Bari, Italy; alessia.catalano@uniba.it

**Keywords:** wheat, natural antimicrobials, polyphenols, antimicrobial peptides, wheat by-products

## Abstract

Background: Antimicrobial resistance (AMR) represents a global health challenge, contributing to elevated rates of morbidity and mortality. This growing problem is attributed to the widespread and indiscriminate use of antimicrobial agents. In response, current research is focused on identifying novel strategies to combat AMR, with particular attention to alternative therapeutic agents. Natural antimicrobials have emerged as promising candidates. Among these, wheat, one of the most cultivated food crops in the world, is identified as a valuable source of such bioactive compounds. Beyond its nutritional importance and prevalent use in food production, wheat is rich in polyphenols, small peptides, benzoxazinoids, 1,4-benzoquinones, and 5-*n*-alkylresorcinols. In vitro investigations have demonstrated that these phytochemicals possess broad-spectrum antimicrobial activities, exhibiting efficacy against Gram–positive and Gram–negative bacteria, as well as various fungi. Methods: Two databases, i.e., Google Scholar and Scopus, were screened using different keywords. Results: A series of key compounds responsible for these effects were identified, evaluating wheat’s potential role as a sustainable source of novel and potent antimicrobial agents. Conclusions: This review aims to collect the latest findings regarding the antimicrobial potential of different wheat varieties and their by-products.

## 1. Introduction

Wheat is one of the most cultivated and consumed cereals in the world and it constitutes a primary source of nutrition. It can provide up to 20% of total caloric intake for the entire population, thanks to the presence of essential nutrients such as carbohydrates, vitamins, and proteins [1]. In addition to this fundamental role in human nutrition, wheat has achieved increasing scientific interest, in recent years, for its nutraceutical properties. Particular attention has been directed toward the numerous bioactive compounds present in the grain and its derivatives and processing wastes, including polyphenols, small peptides, alkylresorcinols, and other secondary metabolites, which have shown significant antioxidant, antiproliferative, anti-inflammatory, and antimicrobial effects [2,3]. In parallel, the scientific community is searching for natural alternatives to synthetic antibiotics, which the overuse of has led to the spread of resistant microbial strains, with serious implications for human health. Indeed, the antimicrobial resistance (AMR) has emerged as a huge public health problem, with a forecast of 10 million deaths per year by 2050 [4], and has been included in many political conference agendas. Indeed, since the discovery of penicillin, over 150 new antibiotics have been developed; however, their inappropriate use has led to the development of multidrug-resistant microbes, called ‘superbugs’ [5,6]. One of the consequences could be a return to a pre-antibiotic era, in which even minor infections can once again become life-threatening.

In particular, the principal mechanisms through which antimicrobial resistance can occur include reduced drug absorption, enzymatic inactivation, or increased efflux of the compounds, as well as structural or functional alteration of the therapeutic targets. This phenomenon occurs not only for intrinsic microbial characteristics, but also for factors associated with healthcare providers and consumers. Among the main contributors are the misuse and/or overuse of antimicrobial drugs in both humans and animals (e.g., antibiotics are used as growth promoters in feed), inappropriate prescribing patterns, limited awareness about conscious use, and a reduced availability of new antibiotics entering the market [7]. Therefore, improper use exposes microbes to sub-inhibitory concentrations of antimicrobials, making this practice the main driver of AMR [8].

In this scenario, plant-derived compounds, in particular those derived from cereals such as wheat, represent a precious and little-exploited resource. Wheat contains a variety of substances with antimicrobial potential, which are distributed not only in the whole grain, but also in the processing by-products, such as bran, germ, and gluten. Scientific evidence highlights how different classes of natural compounds, such as polyphenols and polypeptides, can act against multidrug-resistant (MDR) microorganisms [9,10]. These compounds are also present in wheat and its by-products, highlighting the antimicrobial potential of this cereal as a reservoir of natural antimicrobial agents.

This review aims to provide a detailed overview of the antimicrobial potential of wheat, analysing its composition and the distribution of its main active compounds. Experimental evidence on their efficacy against various pathogenic microorganisms, particularly bacteria and fungi, and proposed mechanisms of action will also be discussed. The intention is to valorise and promote the use of wheat as a sustainable source of functional molecules with high added value.

## 2. Wheat: History, Cultivation, and Global Significance

Wheat is a widely cultivated and globally spread cereal, essential to producing different food derivatives such as flour, pasta, and bread. It is an ancient cereal, belonging to the genus *Triticum* within the Gramineae family, which also includes barley, oats, maize, rice, rye, millets, and sorghum. The earliest types of cultivated wheat were the diploid einkorn and the tetraploid emmer, believed to have originated in south-eastern Turkey. Today, however, about 95% of the cultivated wheat is hexaploid, because of the species’ evolution. This crop, scientifically known as *Triticum aestivum*, is mainly used for bread making. The remaining 5% of the world’s cultivated wheat is the tetraploid *Triticum durum*, which is used in pasta production. Other species include emmer, spelt, and einkorn, which are less cultivated [11]. In the marketing year of 2024/25, the total global production volume of wheat amounted to 793 million metric tonnes, with a global per capita food use of wheat of 63.9 kg/year [12]. Indeed, this cereal holds particular importance in the human diet, due to its nutritional properties, which are correlated to its chemical and physical composition.

### Structure and Chemical Composition of Wheat Grain

**Figure 1:**
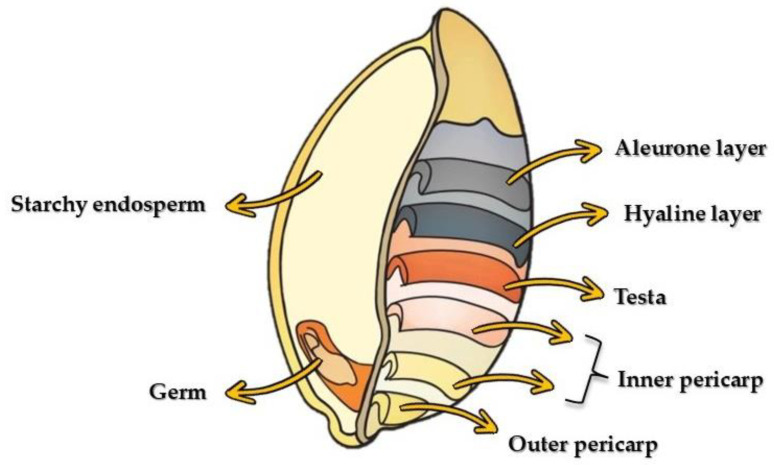
Structure of wheat grain.

The wheat grain is often incorrectly defined as a seed, whereas it is a fruit, more precisely a caryopsis, characterised by a single seed, which is closely connected to or entirely fused with the wall of the mature ovary (pericarp) [13]. From a morphological perspective, wheat grain presents an oval shape, with a well-defined longitudinal furrow. It generally does not exceed 10 mm in length and 4.5 mm in width [13] and it is possible to distinguish three main sections: the germ (also known as embryo), the endosperm, and the outermost layers, collectively referred to as the bran. The germ constitutes 2–3% of the grain’s weight and is rich in lipids and fat-soluble vitamins, including E vitamin, and B vitamins. The endosperm, on the other hand, accounts for 80–85% of the total grain’s weight and consists mainly of protein, starch, and a low fibre content (2%). Surrounding the endosperm is the aleurone layer, which contains significant bioactive molecules, such as polyphenolic compounds, lignans, as well as vitamins and minerals. The bran consists of the hyaline layer (or nucellar epidermis), testa (which is rich in alkylresorcinols), and inner and outer pericarp (which have high levels of xylans, cellulose, and lignin). It generally constitutes about 12–18% of the grain’s weight (Figure 1) [11,14].

As reported in a recent study [15], common wheat and durum wheat exhibit approximately similar contents of carbohydrates (~73%), lipids (~2%), proteins (~12%), and fibres (~12%), as well as excellent levels of essential minerals such as magnesium, phosphorus, potassium, and calcium. The polysaccharides, which are mainly present in the cell wall, include arabinoxylans (structurally linear, whose monomeric unit is β-D-xylopyranose) and β-glucans (consisting of β-D-glucose units with β-1,3 and β-1,4 bonds). It is also interesting to highlight the polyphenol content within the wheat grain. Polyphenols, known for their beneficial properties, are secondary metabolites produced in response to environmental stress (e.g., climate, parasites) or as a result of physiological stimuli. The most representative in wheat is ferulic acid, which is commonly found together with *p*-coumaric, sinapic, vanillic, and caffeic acids. These phenolic acids are often present in esterified form with arabinoxylans or conjugated with oligosaccharides [16] and they are typically located in the bran and germ of the wheat grain, which are often considered by-products in the food industry, during the refinement process to produce flour and derivatives [17]. The phytochemical composition of the wheat grain is in any case highly variable, obviously influenced by cultivar type, genetic variability, environmental conditions, and agronomic practices [18]. Nonetheless, the presence of polyphenols, alkylresorcinols, carotenoids, and vitamins confer wheat a nutraceutical potential that cannot be underestimated.

## 3. Antimicrobial Resistance and Natural Antimicrobials

Antibiotic resistance is closely linked to high morbidity and mortality rates, due to multidrug-resistant patterns observed in both Gram–positive and Gram–negative bacteria. The overuse and misuse of broad-spectrum antibiotics exacerbate this issue, leading to the ineffectiveness of conventional antimicrobials over time [19]. Furthermore, the improper use of antibiotics in animal husbandry and the food industry is a contributing factor to this phenomenon [20]. Indeed, in response to high consumer demand, the pace of production and transport has increased in this sector, along with the risk of contamination; indeed, various microorganisms are often detected in food, including *Listeria monocytogenes*, *Escherichia coli*, *Staphylococcus aureus*, and *Salmonella*, necessitating the use of antimicrobials in order to limit infections and prevent the resulting clinical manifestations [21]. In this context, the World Health Organization (WHO) released the Bacterial Priority Pathogens List (BPPL) in 2024, establishing priority levels for antibiotic-resistant bacteria in order to guide global attention and research efforts [22]. Within this list, bacterial strains are classified into three categories based on low, medium, or high priority to encourage therapeutic development and public health interventions. The 2024 WHO BPPL covers 24 pathogens. Notably, among them, *Klebsiella pneumoniae*, *Escherichia coli*, *Acinetobacter baumannii*, *Salmonella Typhi*, *Shigella* spp., *Pseudomonas aeruginosa*, *Staphylococcus aureus*, *Streptococcus pneumoniae*, *Enterobacter* spp., *Proteus* spp., and *Serratia* spp. stand out, whose inhibition will be examined in the following sections.

Additionally, the antifungal resistance is another growing issue, i.e., an emerging resistance to the few classes of antifungal drugs available, driven by genetic factors (global transcription and chromosomal factors that induce resistant phenotypes) and by the application of agricultural fungicides [23].

Considering this, it is necessary to explore viable alternatives to the use of conventional antibiotics and antimycotics, minimising the risk and/or incidence of antimicrobial resistance. One valid strategy could be the use of antimicrobials, derived from natural sources, which are often identified in various plant components, including roots, leaves, seeds, flowers, fruits, etc. They have been shown to possess not only excellent antibacterial properties, but also antifungal activity. The molecules responsible for these activities include phenolic acids, flavonoids and flavone glycosides, coumarins, terpenoids, lignans, polysaccharides, organic acids, saponins, thiosulfinates, essential oils, and peptides [21,24]. Some of these natural antimicrobials have also been found in wheat, with proposed mechanisms of antimicrobial action such as altered permeability of microbial cells, as well as electron delocalisation promoted by hydroxyl groups, leading to a decay of the proton motive force and, consequently, cell death. Moreover, they may interfere with nucleic acid replication and/or transcription [24]. The potential applications of natural antimicrobials could be manifold: in the food sector (food packaging), in cosmetics (acne treatment) and, especially, in the pharmaceutical field. Clearly there could be limitations, especially regarding safety concerns, but the potential of natural antimicrobials remains promising.

## 4. Antimicrobial Compounds in Wheat

As already reported, common wheat has been demonstrated to be a rich source of different bioactive compounds (e.g., polyphenols, lignans, carotenoids, tocopherols and tocotrienols, alkylresorcinols, dietary fibre, and resistant starch), which are responsible for numerous pharmacological activities, including anti-diabetic action and positive impacts on metabolic diseases, antioxidant activity, cholesterol-lowering effect, as well as anti-cancer and anti-inflammatory ones [25,26]. Recently, several studies have also investigated the antibacterial and antifungal properties of wheat grains. Such activities are attributed not only to the presence of polyphenols, whose structural diversity is the basis of their broad spectrum of antimicrobial targets [27], but also to peptides and various secondary metabolites recognised for their beneficial health properties, many of which are present in wheat by-products too.

### 4.1. Polyphenols

**Figure 2:**
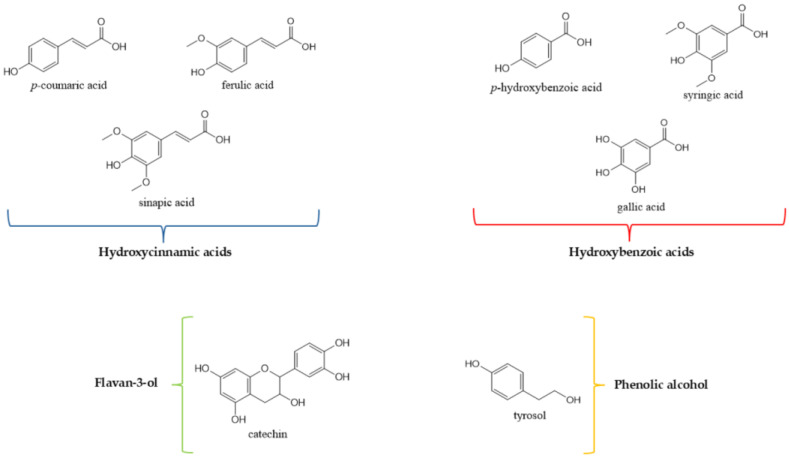
Chemical structures of phenolic compounds found in wheat that exhibit antimicrobial activity.

**Figure 3:**
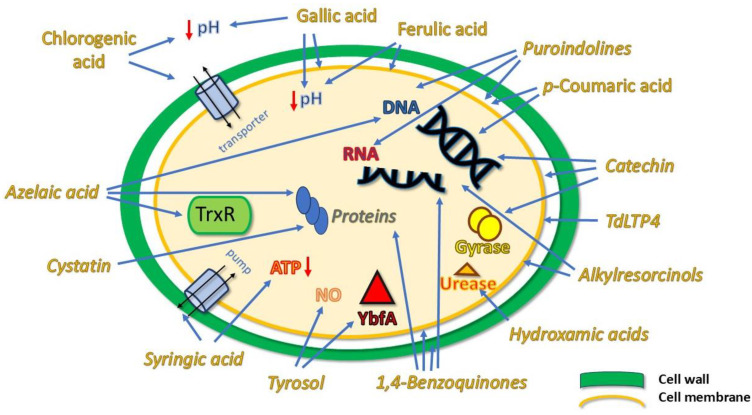
Cartoon depicting the main bacterial targets on which wheat components may act.

Polyphenols are secondary metabolites produced by plants, found in various fruits, vegetables, cereals, as well as in their derivatives such as juices and both alcoholic and non-alcoholic beverages. These compounds can be classified, according to their chemical structures, into flavonoids, phenolic acids, lignans and lignins, stilbenes, tannins, xanthones, chromones, and anthraquinones. Phenolic acids, in particular, can be further subdivided into hydroxybenzoic and hydroxycinnamic acids (Figure 2) [28,29] and have long been recognised for their excellent antimicrobial activities. Generally, the phenolic acid’s antimicrobial activity depends on the considered bacterial species and strain, but also the chemical structure of the compounds (Figure 3) [30]. A recent study have confirmed these findings, testing the antimicrobial activity of a particular hydroxycinnamic acid, *p*-coumaric acid [31]. The latter (Figure 2) is one of the predominant phenolic acids in cereals, especially in the wheat pericarp, and it exhibits considerable inhibitory activity against Gram–positive bacteria, such as *Bacillus cereus* and *Bacillus subtilis*, as well as Gram–negative bacteria *Salmonella typhimurium* and *Shigella dysenteriae*. The antibacterial activity may be due to an alteration of cell membrane permeability and disruptions of cell functions, attributed to the lower number of hydroxyl groups compared to hydroxybenzoic acids. One of the proposed mechanisms is the ability to decrease the extracellular pH, as for gallic and chlorogenic acids, altering cell membrane potential and affecting the solutes transport [32]. The acidification of the phenolic acids, as for gallic and ferulic acids, can also happen intracellularly, decreasing the cytoplasm pH value and disrupting, as well in this case, the cell membrane structure and causing protein denaturation (Figure 3) [33].

Jeong et al. [34] produced three extracts derived from wheat (*Triticum aestivum* L.) sprouts, collected from the National Institute of Crop Science, Rural Development Administration (RDA). These extracts, obtained using ethanol, methanol, and water, respectively, were tested for antimicrobial activity against oral bacteria employing the paper-disc agar-diffusion method. In particular, the water extract exhibited strong growth inhibition of *Actinomyces viscosus* and *Streptococcus salivarius* at a dose of 20 mg/disc, while the alcohol extract, at the same dosage, showed moderate inhibition, suggesting that polarity in the extractive field influences such activities, which could be further investigated Through a chemical analysis, the presence of benzoic, *p*-hydroxybenzoic, *p*-coumaric, ferulic, and sinapic acids was revealed. Consequently, the antimicrobial activity of these compounds was also tested individually, finding that ferulic acid, *p*-coumaric, and *p*-hydroxybenzoic acids (Figure 2) are primarily responsible for the observed property, supporting the hypothesis that polyphenols have a significant antimicrobial potential. Furthermore, it has been demonstrated that *p*-coumaric acid exerts its bactericidal activity interfering with cell membranes, binding the phosphate groups and intercalating into the genomic DNA. In this way, it affects the replication, transcription, and expression processes (Figure 3) [35].

It is well-established that following a massive pathogenic infestation of grains, there is an increase in the biosynthesis of phenolic compounds, to potentiate the plant’s own defensive capacity. Przybylska-Balcerek et al. [36], therefore, inoculated microscopic fungi from the genus *Fusarium* (including *F. culmorum Sacc.* (two isolates), *F. graminearum Schwabe* (2 isolates), and *F. langsethiae* (Torp & Nirenberg (isolate 8051)) into wheat and test their antimicrobial activity, following chemical characterisation. The correlation between the minimum inhibitory concentration (MIC) and the content of ferulic acid and sinapic acids (Figure 2) was found to be significant. The obtained extracts demonstrated bactericidal activity against *Micrococcus luteus*, *Pseudomonas fluorescens*, *E. coli*, and *Proteus mirabilis*, as well as antifungal activity against *F. culmorum*, *F. graminearum*, and *F. langsethiae*, with MIC values ranging from 0.20 to 2.86 µg/g extract. Suchowilska et al. [37] have previously carried out a similar study on different common wheat varieties. Following the infestation by *F. culmorum*, an increased resistance was observed in the varieties Torka and Zebra, which exhibited a high polyphenol content (1236.58 µg/g). The resulting methanolic extracts have been shown to inhibit the growth of *F. culmorum*, which can be correlated with the concentration of phenolic compounds. Therefore, polyphenols play a role in the expression of certain types of resistance to this fungus.

A valid approach to enhance the nutritional and bioactive properties of cereal grains is their fermentation with beneficial lactic acid bacteria, such as *Lactiplantbacillus plantarum*. The aqueous extract of modern-day hard red spring wheat was tested by the agar-diffusion method against *Helicobacter pylori*, the bacterium responsible for gastric ulcer outbreaks. The detected antimicrobial activity was correlated with low pH conditions, in combination with lactic acid and phenolic compounds such as catechin, gallic acid, and ferulic acid (Figure 2). Furthermore, these extracts showed no negative impact on beneficial gut microbes, such as *Bifidobacterium longum*, suggesting that fermentation can be a valid strategy to target and manage pathogenic bacteria [38]. It is known that catechins and other flavonoids possess anti-Gram–positive and –negative bacterial activity; they can enter the lipid bilayer causing bacterial cell membrane disruption or fusion, resulting in intramembranous components leakage. They can also inhibit nucleic acids metabolism by DNA intercalation and gyrase inhibition (Figure 3) [39].

Finally, excellent antimicrobial properties were also demonstrated by flours derived from both ancient and modern wheat varieties. Grande et al. [40] produced hydroalcoholic extracts (Ethanol 80%) from four ancient wheat varieties (Ostro nudo, Antigola, Saragolla, and Primitivo) and four modern wheat varieties (Palesio, Bolero, Bologna, and Rebelde), all grown in Italy. The main phenolic compounds identified were resorcinol, tyrosol, and syringic acid (Figure 2), and bactericidal activity against both Gram–positive and Gram–negative bacteria was evaluated. For example, tyrosol was found to inhibit the *E. coli* protein YbfA, which mediates the anaerobic biofilm formation and modulates NO (nitric oxide) synthesis [41]. Syringic acid perturbs cell membrane permeability inducing ion leakage and proton influx, and can decrease ATP concentrations and alter intracellular pH (Figure 3) [42]. The growth inhibition of *E. coli* (at 0.19 mg/mL) and *Enterococcus faecalis* (also at 0.19 mg/mL) was more pronounced in the extracts obtained from Palesio, a result that was confirmed by the individual testing of ferulic acid. Additionally, an induction of bacterial growth was observed in *Lactobacillus brevis*, a probiotic bacterium, especially by Rebelde and Primitivo. Therefore, the performance of these activities could be influenced by factors such as cultivation methods and environmental conditions.

These findings suggest that polyphenols in wheat possess excellent bactericidal and fungicidal properties (Table 1), which can also be implemented through fermentation. Such activities are also evidenced in derivatives such as flours, although further investigation and confirmation are necessary. Polyphenols contained in wheat can therefore be considered as potential natural antimicrobials. Indeed, while the polyphenols in question have been shown to exhibit the aforementioned antimicrobial properties, these are often examined at concentrations that cannot be achieved physiologically through diet. It should also be clarified whether these natural compounds act individually or synergistically. Nevertheless, polyphenols found in wheat can be considered potential natural antimicrobials.

### 4.2. Antimicrobial Peptides

Antimicrobial peptides (AMPs) are regarded as promising therapeutic agents, due to their strong activity and low possibility of resistance development by many pathogens. These peptides are small molecules, consisting of a few amino acid residues (<100), which are synthesised in plants as a part of their defence mechanisms. AMPs are mainly amphipathic or cationic (due to the presence of arginine and lysine residues), with a smaller fraction being anionic [43,44]. They can act as signalling molecules or immunomodulators and have also been found in wheat. Dhatwalia et al. [45] were among the first to isolate a series of proteins, called puroindolines, from the endosperm of the wheat grain by Triton X-114 phase partitioning. They highlighted the growth inhibitory activity of these proteins against pathogens such as *S. aureus*, *Micrococcus lutius*, *Klebsiella*, and *Bacillus circus* at low concentrations (<1 mg/mL). Puroindolines share many characteristics with other antimicrobial proteins but differentiate for a conserved cysteine backbone [46]. Capparelli et al. [47] further confirmed the antimicrobial potential of these native wheat-derived proteins. In particular, they tested the inhibitory effect on various Gram–positive and Gram–negative bacterial strains, such as *Agrobacterium tumefaciens*, *Clavibacter michiganensis*, *E. coli*, *Erwinia carotovora*, *Pseudomonas syringae*, and *S. aureus*. The MICs ranged from 30 to 50 μg/mL, with later studies proving these findings, reporting a growth inhibition of 40–50% against *E. coli* and *S. aureus* [48]. As well as for puroindolines, the main bacterial targets were disclosed to be cell membrane and nucleic acids (DNA and RNA) (Figure 3) [49,50].

Another notable class of polypeptides is cystatins, which usually exhibit inhibitory activity against cysteine proteinases, in which resides the antibacterial activity, since the proteolytic activity is necessary for bacterial growth (Figure 3) [51]. Known for their insecticidal activity, cystatins have also been isolated from wheat. In particular, Christova et al. [52] isolated a multidomain cystatin, TaMDC1, which is over-expressed in wheat during cold acclimation, probably as a defence system against winter pathogens. This gene expression is also up-regulated in response to treatment with methyl jasmonate and salicylic acid, suggesting a role of cystatin in biotic stress responses. Transgenic tomato plants, expressing TaMDC1, demonstrated enhanced resistance to *P. syringae* bacteria and the pathogenic fungi *Botrytis cinerea* and *Alternaria alternata*, showing significantly fewer leaf lesions compared to controls.

Pathogenesis-related proteins (PR-14) also play a crucial role in plant defence mechanisms. Lipid transfer proteins (LTP) are members of this family and recently, Ben Hsouna et al. [53] cloned a new gene, TdLTP4, from *Triticum turgidum*, which encodes an antifungal and antibacterial protein. The inhibition zone diameter, MIC, minimum bactericidal concentration (MBC), and minimum fungicidal concentration (MFC) were determined using different Gram–positive bacteria and Gram–negative bacteria, as well as fungi. The results revealed that the TdLTP4 protein exhibited a significant antibacterial effect against *S. aureus* and *L. monocytogenes*, along with a good antifungal activity against *Fusarium oxysporum* and *F. graminearum*, with an MIC of 62.5 μg/mL. The mechanism by which TdLTP4 protein acts resides in the disruption of the microbial membranes, transporting lipids into microbial cells (Figure 3) [54]. Additionally, some AMPs have been identified in wheat seeds (*Triticum durum*), which Talas-Ogras [55] tested for their antimicrobial activity. Using SDS-PAGE, he analysed the purity of small protein fractions, obtained from both germinated and non-germinated wheat seed meal. A transparent zone of inhibition was observed around discs containing protein samples, indicating antifungal activity, especially against *Rhizoctonia solani*. However, the peptides did not influence the growth of the Gram–negative bacteria. A detailed summary of the studies conducted is provided in Table 1.

**Table 1:** A detailed summary of studies on the antimicrobial efficacy of polyphenols and peptides in wheat.

Wheat Variety	Bioactive Compounds	Target Microorganisms	Findings	Reference
Not available	*p*-coumaric acid	*Bacillus cereus*, *Bacillus subtilis*, *Salmonella typhimurium*, and *Shigella dysenteriae*	MIC values ≈ 80 µg L^−1^	[31]
Not available	Benzoic, *p*-hydroxybenzoic, *p*-coumaric, ferulic, and sinapic acids	*Actinomyces viscosus*, *Lactobacillus rhamnosus*, *Streptococcus mutans*, *Streptococcus salivarius*, and *Streptococcus sobrinus*	Inhibition zone diameters ranged from 10–15 mm to 21–30 mm at doses between 1 and 5 mg/disc	[34]
Astoria, KWS Ozon, Kandela, and Torka	Ferulic and sinapic acids	Bacteria: *Escherichia coli*, *Pseudomonas fluorescens*, *Micrococcus luteus*, and *Proteus mirabilis* Fungi: *Fusarium culmorum*, *Fusarium graminearum*, and *Fusarium langsethiae*	MIC values ranged from 0.20 to 2.86 µg/g extract. Lowest MIC values observed for *Fusarium langsethiae* 8051	[36]
Kontesa, Torka, Hena, Helia, and Broma	Phenolic compounds	*Fusarium culmorum*	Treated cultures exhibited surface areas between 27.41 and 31.30 cm^2^, compared to 42.01 cm^2^ in control	[37]
North Dakota Common Emmer and hard red spring wheat cv. Barlow	Benzoic, gallic, protocatechuic, and ferulic acids and catechin	*Helicobacter pylori*	Inhibition zones observed at 72 h, ranging from 2 to 4 mm in width	[38]
Ancient wheat varieties: Ostro nudo, Antigola, Saragolla, and Primitivo Modern wheat varieties: Palesio, Bolero, Bologna, and Rebelde	Resorcinol, tyrosol and caffeic, syringic, and ferulic acids	*Escherichia coli*, *Salmonella enterica* ser. *Typhimurium*, *Enterobacter aerogenes*, *Staphylococcus aureus*, and *Enterococcus faecalis*	The final optical density was significantly reduced at 630 nm after incubation with different sample extracts (0.19, 0.39, 1.56, 4.68 mg/mL)	[40]
Not available	Puroindolines	*Staphylococcus aureus*, *Micrococcus lutius*, *Klebsiella*, and *Bacillus circus*	Zone of inhibition ranged from 2 to 20 mm. Decreased protein concentration (from 3 mg/mL to 1 mg/mL) led to reduced inhibition zones	[45]
Not available	Puroindolines	*Agrobacterium tumefaciens*, *Clavibacter michiganensis*, *Escherichia coli*, *Erwinia carotovora*, *Pseudomonas syringae*, and *Staphylococcus aureus*	MICs ranged from 30 to 50 μg/mL	[47]
Not available	Puroindolines	*Escherichia coli*, *Serratia marcenscens*, and *Staphylococcus aureus*	At 1 mg/mL, the protein inhibited approximately 40–50% of microbial growth for all three species	[48]
*Triticum aestivum* L. cv. Chihoku komugi	Wheat multidomain cystatin (TaMDC1)	Bacteria: *Pseudomonas syringae* Fungi: *Botrytis cinerea and Alternaria alternata*	All TaMDC1-expressing plants displayed marked reduction in lesion size as compared to control plants	[52]
Not available	Wheat lipid transfer protein (TdLTP4)	Bacteria: *Bacillus subtilis*, *Bacillus cereus*, *Staphylococcus aureus*, *Staphylococcus epidermis*, *Enterococcus faecalis*, *Listeria monocytogenes*, *Salmonella enterica*, *Escherichia coli*, and *Pseudomonas aeruginosa.* Fungi: *Aspergillus niger*, *Aspergillus flavus*, *Aspergillus nidulans*, *Aspergillus fumigatus*, *Fusarium graminearum*, *Fusarium oxysporum*, *Fusarium culmorum*, and *Alternaria alternata*.	Zone of inhibitions ranged from 14 to 26 mm, while MIC values were 62.5 µg mL^−1^ against *Staphylococcus aureus*, *Fusarium graminearum*, and *Listeria monocytogenes.* Other MIC values ranged from 125 to 250 µg mL^−1^	[53]
*Triticum durum* L. cv. Altintoprak-98	Wheat Antimicrobial Peptide (WAP) and Germinated Wheat Antimicrobial Peptide (GWAP)	Fungi: *Botrytis cinerea*, *Fusarium oxysporum*, *Rhizoctonia solani*, and *Verticillium dahlia* Bacteria: *Clavibacter michiganensis* subsp. *sepedonicus* and *Erwinia carotovora* subsp. *carotovora*	IC_50_ values of WAP and GWAP against fungi ranged from 20 to 40 μg cm^−3^ and 15 to 30 μg cm^−3^, respectively GWAP inhibited the growth of *Clavibacter michiganensis* at 180 μg protein cm^−3^	[55]

Overall, although the antimicrobial potential of wheat-derived AMPs has been confirmed, the selected literature is largely based on in vitro assays only. The conditions under which these assays are carried out limit direct comparison. Furthermore, the stability and bioavailability of these peptides, which show promise as therapeutic agents, should be investigated.

### 4.3. Other Antimicrobial Compounds in Wheat

The presence of polyphenols and small peptides in wheat has spurred the search for bioactive antimicrobial compounds in extracts derived from various parts of wheat plant, including the leaf, stem, root, awn, and seed. Saha et al. [56] reported the susceptibility of *E. coli* and *S. aureus* to extracts from wheat seed, awn, and root prepared using both methanolic and ethanolic solutions. However, the demonstrated antibacterial effects are not always exclusively attributable to polyphenols or small peptides. Extensive research has also suggested that antimicrobial action may be linked to other classes of secondary metabolites present in the wheat plant.

**Figure 4:**
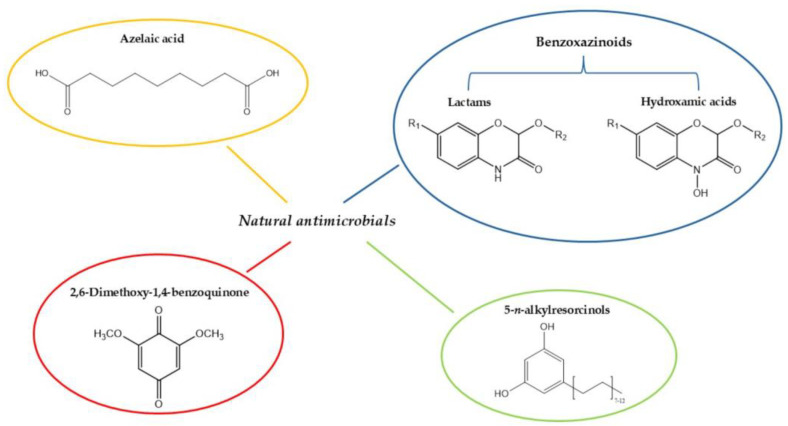
Chemical structures of compounds found in wheat that exhibit antimicrobial activity.

One example is the antibacterial activity of azelaic acid (Figure 4). The latter is a saturated C9 dicarboxylic acid, produced by wheat in response to some stress conditions, including both biotic and abiotic stress. Azelaic acid is typically used in topical treatments for mild to moderate acne, due to its ability to reduce inflammation, coupled with its antibacterial effect. Azelaic acid inhibits the bacterial oxidoreductase enzymes (e.g., thioredoxin reductase-TrxR) responsible for microbial respiration, protein, and DNA synthesis (Figure 3) [57]. Spaggiari et al. [58] utilised different extraction techniques for isolating azelaic acid from wheat, with the Naviglio method proving to be the most efficient. Their study demonstrated that this compound exhibited antibacterial activity against *Streptococcus pyogenes* at a concentration of 256 μg/mL.

Even 1,4-benzoquinones (Figure 4) are known for their antimicrobial properties. Kim et al. [59] characterised a series of these compounds in wheat germ. Using HPLC analysis, they identified natural quinones that exhibited antibacterial activity against *S. aureus* and *B. cereus*, with inhibition zone diameters of 27.6 mm and 17.8 mm, respectively. In particular, 2,6-dimethoxy-1,4-benzoquinone was the most representative quinone in the wheat extracts. Its antimicrobial activity was evaluated against other benzoquinones, revealing an interesting structure-dependent activity, where methoxylated compounds exhibited enhanced effect. Interestingly, however, this trend was opposite for Gram–negative bacteria, where methoxylation leads to a reduction in antimicrobial activity, suggesting that modifications affecting the molecule’s hydrophobicity may determine its interaction with the membrane, an aspect that has not been sufficiently investigated in the literature. The main mechanisms proposed for 1,4-benzoquinones antibacterial activity are the interference with cell membrane function and cell wall, protein, and nucleic acids synthesis (Figure 3) [60].

Recently, benzoxazinoids have been identified not only in young wheat plants, but also in wholegrain products. These compounds, chemically categorised in the hydroxamic acids and lactams (Figure 4), were found to be active against *S. aureus*, *E. coli*, and *Candida albicans*, with the substituted C6 position being critical for their antimicrobial activity [61]. The compound 6-methoxy-benzoxazolin-2-one surprisingly demonstrated an inhibitory effect on human immunodeficiency virus (HIV) reverse-transcriptase activity, with 60% inhibition at concentrations of 800 μM [62], while the microbially transformed derivative 2-amino-3*H*-phenoxazin-3-one has also shown interesting tuberculostatic and antimicrobial activity against *Mycobacterium phlei*, *Penicillium chrysogenum*, *Monilia Formosa*, and *Schizosaccharomyces actosporas* at concentrations of 2 μg/mL [63]. Furthermore, Bravo et al. [64] isolated specific hydroxamic acids (derived from 2,4-dihydroxy-1,4-benzoxazin-3-one) in wheat extracts and evaluated their minimum inhibitory concentration for microbial growth in vitro, including *S. aureus*, *E. coli*, and the fungus *C. albicans*. The MIC values were generally found to be 333 µg mL^−1^ and the antimicrobial activity was linked to the specific inhibition of microbial urease, probably due to the interaction between specific hydroxamic acid with the metal ion present in the enzyme’s active site (Figure 3). This study also demonstrated that the acyclic form of the hydroxamic acid structure is nonetheless active, suggesting that the hemiacetal at the C-2 position is not essential for the antimicrobial activity.

Among the various compounds found in wheat there are the 5-*n*-alkyl(enyl)resorcinols, which belong to the class of lipid phenols (Figure 4). Structurally, these molecules are composed of a meta-dihydroxy-substituted benzene ring with an alkyl or alkenyl side chain at the C-5 position [65]. It is generally established that alkylresorcinols are abundant in the intermediate layers of the wheat grain (hyaline layer, integument and intermediate pericarp), although they are also in wheat derivatives. Indeed, Marentes-Culma et al. [66] reported a high total content of 5-*n*-alkyl(enyl)resorcinols in wheat flour and the acetone extract of this matrix inhibited the mycelial growth of *F. oxysporum* by 77.2% at a concentration of 10.0 μg/μL. In addition to their antifungal properties, these compounds also demonstrate a good antibacterial activity against Streptococcus mutans, an etiological agent of paradonthosis and *Propionibacterium acne*, the bacterium responsible for acne. In fact, homologues, containing unsaturated chains, have been shown to possess MIC values ranging from 0.78 to 1.56 μg/mL. Interestingly, the strong antifungal activity detected in flour extracts suggests that alkylresorcinols persist after milling process. Generally, the antibacterial activity of alkylresorcinols is based on the structural modification of cell membranes, increasing the viscosity and compromising their functional activities, together with the interference with DNA and metabolising enzymes (Figure 3) [67].

Finally, long-chain 5-*n*-alkylresorcinol homologues also have shown molluscicidal activity against *Briomphalaria glabratus*, a parasite causing schistosomiasis (a tropical disease). Among the resorcinol lipids, the most active agent was 5-*n*-pentadecenylresorcinol [68], the chemical structure of which is shown in Figure 4. The molluscicidal activity of long-chain homologues extends the biological spectrum of alkylresorcinols further, although the scope of these results is currently limited.

## 5. Antimicrobial Properties of Wheat By-Products

Approximately 150 million tons of wheat by-products are generated annually. This is due to flour extraction rate, which is between 73% and 77%, with the remaining fraction involving wastes such as wheat germ, wheat bran, and parts of the endosperm. Fortunately, these by-products are being repurposed. Wheat germ, for instance, is often introduced into food formulations, such as snacks or bread. Its oil content ranges from 8% to 14% (depending on the wheat variety) and it is also exploited in medical and cosmetic applications [69]. Notably, wheat germ oil, which is rich in phytosterols and tocopherols, has demonstrated excellent antibacterial and antifungal properties, through the disc diffusion method [70]. It has been shown to inhibit the growth of *E. coli* (8.3 mm), *S. aureus* (10 mm), *Klebisiella pneumoniae* (8.7 mm), and *Acinetobacter baumannii* (8.7 mm), all of which are Gram–negative bacteria. Most importantly, it exhibited an excellent antifungal effect against *C. albicans*, with a zone of inhibition measuring 11.67 mm. Conversely, defatted wheat germ extracts prepared by Mahmoud et al. [69] showed better antibacterial activity against Gram–positive strains, especially *L. monocytogenes*. It is plausible that the limited efficacy of the extract against Gram–negative bacteria is attributable to the presence of multidrug resistance pumps or the high lipid content of their outer membrane, which is not found in Gram–positives.

Wheat bran, another waste product in the wheat industry is a source of bioactive molecules. In addition to its high content of polyphenols, it contains several AMPs that exhibit excellent antibacterial properties. Zou et al. [71] isolated three peptides from wheat bran using ultrafiltration, with different molecular weights (<3 kDa, 3–5 kDa, and 5–10 kDa). They exhibit the lowest MICs of 4, 5, 3, and 2.5 mg/mL against *E. coli*, *S. typhimurium*, *S. aureus*, and *L. monocytogenes*, respectively. Through RP-HPLC, the <3 kDa fraction was separated into four fractions (F1–F4), among which the F2 fraction showed the highest antimicrobial activity. From this fragment, a small peptide named WBp-1 (with the amino acid sequence IITGASSGIGKAIAKHFI) was subsequently identified and further studies revealed that it exerts its antibacterial effect by compromising the structural integrity of the *L. monocytogenes* cell membrane, via interaction with the amino acids of the peptidoglycan synthase PBP4. The identification of WBp-1 provides an important proof of concept for the presence of potent AMPs in wheat bran; however, its activity occurs at relatively high concentrations compared to classical AMPs.

Recently, Ramadan et al. [72] also focused on wheat bran valorisation. Under solid-state fermentation conditions, using *Aspergillus pseudodeflectus*, they obtained the production of methyl ferulate and oleic acid from wheat bran. Both compounds exhibited significant antibacterial potential against Gram–positive bacteria, especially *B. subtilis* and *S. aureus*, with MIC values of 79% and 69%, respectively.

Finally, the reuse of wheat gluten, a by-product of the wheat starch industry, is notable. Gluten is a protein composed by gliadins and glutenins, which undergoes hydrolysis, resulting in some peptides that have drawn scientific attention [73]. Using ficin, a hydrolytic enzyme, it is possible to obtain peptides (wheat gluten protein hydrolysates obtained after 60 min (WGPHT1), 120 min (WGPHT2), and 180 min (WGPHT3) of hydrolysis) showing antibacterial activity against *S. aureus* and *E. coli*, with an MIC of 50 mg/mL. This could be an opportunity to valorise a by-product, reusing it to obtain small AMPs that could be included into food formulations and healthcare products. It is important to note that, generally, these protein fragments are inactive in the parent protein molecules; however, once released through hydrolysis, for example, they exert various biological and functional effects, like antimicrobial activity. Therefore, these bioactive compounds may be a novel alternative to traditional antibiotics. In the context of the circular economy, the conversion of gluten into bioactive peptides is an interesting approach, provided its stability and safety are assessed.

## 6. Wheatgrass

Wheatgrass is a tender shoot derived from wheat. Recently, it acquired considerable attention due to its potent antioxidant and antimicrobial activities, related to the presence of vitamins E and C, β-carotene, ferulic, and vanillic acids [74]. Das et al. [75] prepared extracts from freeze-dried wheatgrass using an 80% methanol solution and reported excellent antibacterial capacity against *Aspergillus niger* and *E. coli*, with growth inhibition zones of 8.03 and 7.31 mm, respectively. Additional studies have reported significant activity against *Salmonella Enteritidis*, with a 50 mg/mL concentration of wheatgrass extract producing a 7 mm zone of inhibition [76]. Sandersan et al. [77], on the other hand, reported better antibacterial effects of aqueous extract against *Salmonella typhi*, *Streptococcus pneumoniae*, and *L. monocytogenes*. Overall, the antimicrobial activity of wheatgrass, particularly when harvested at an early growth stage, demonstrates its potential as a natural preservative agent. The antimicrobial effect against *L. monocytogenes* was also exerted by wheatgrass juice, as reported by Pehlivanoğlu et al. [78]. Using a fruit squeezer, they realised that such extract determined a growth inhibition zone of 11 mm at a concentration of 1.0 mg/mL. These findings highlight the promising antibacterial properties of wheatgrass, although the efficacy appears to be influenced by the harvesting period and the type of solvent used for the extraction, suggesting that optimisations of these parameters are needed in order to achieve an improvement in antimicrobial activity. Such enhancements could potentially extend its effectiveness against a larger range of microbial targets, including fungi and viruses.

## 7. Methodology

A comprehensive literature search was performed to identify studies specifically focusing on natural antimicrobial agents present in wheat and its by-products. The search was conducted using two major academic databases, Google Scholar and Scopus, without applying any temporal limitations. Different keywords were employed for the search including: “natural antimicrobials in wheat”, “bioactive compounds in wheat”, “antibacterial activity of wheat extracts”, “antifungal activity of wheat extracts”, and “natural antimicrobials in wheat by-products”. The most relevant studies reporting the antibacterial and antifungal activity of extracts from different wheat varieties, or their by-products, published in the English language were selected for preparing this review.

## 8. Conclusions

In light of the scientific evidence reviewed, wheat is confirmed as a significant resource of bioactive compounds with notable antimicrobial potential. Polyphenols, antimicrobial peptides, and various secondary metabolites, present in different components of wheat grain and its processing by-products, show promising activities against a wide range of pathogenic bacteria and fungi. These compounds, like phenolic acids, azelaic acid, alkylresorcinols, TdLTP4, etc., contribute to the plant’s natural defence mechanisms and offer interesting ideas for therapeutic and industrial applications. In particular, inhibitory activity has been observed against a wide range of pathogens, including bacteria, both Gram–positive and –negative, as well as different fungal species.

The identification and characterisation of these bioactive molecules open new perspectives for the valorisation of wheat and its derivatives in the nutraceutical, pharmaceutical, and agro-food fields. In particular, the reuse of wheat processing by-products, such as bran, germ, and gluten, represents a concrete opportunity for the development of sustainable solutions with a view to a circular economy.

Despite the encouraging results, further studies are needed to elucidate the molecular mechanisms of action, optimise extraction and application methods and assess the safety of these compounds in real-world settings. Future research should address the actual bioavailability of these compounds and, where necessary, enhance it through advanced delivery technologies. Simultaneously, the development of structural analogues could facilitate the elucidation of structure–activity relationships and potentially increase their antimicrobial properties. Finally, a comprehensive review of the literature highlights a notable gap regarding the assessment of antiviral activities and, more importantly, the implementation of additional in vivo studies.

Nevertheless, the antimicrobial potential expressed by wheat represents an important resource to be explored, especially in an era where antibiotic resistance is forcing a reassessment of traditional therapeutic strategies.

## Data Availability

No new data were created or analyzed in this study. Data sharing is not applicable to this article.

## Abbreviations

The following abbreviations are used in this manuscript:

AMR	Antimicrobial resistance
RDA	Rural Development Administration
MIC	Minimum inhibitory concentration
TaMDC1	Wheat multidomain cystatin 1
TdLTP4	Wheat lipid transfer protein 4
WAP	Wheat Antimicrobial Peptide
GWAP	Germinated Wheat Antimicrobial Peptide
AMPs	Antimicrobial peptides
PR-14	Pathogenesis-Related Protein-14
LTP	Lipid transfer proteins
MBC	Minimum bactericidal concentration
MFC	Minimum fungicidal concentration
SDS-PAGE	Sodium Dodecyl Sulphate-Polyacrylamide Gel Electrophoresis
HIV	Human immunodeficiency virus
RP-HPLC	Reversed Phase-High-Performance Liquid Chromatography
WBp-1	Wheat Germ Agglutinin-Binding Protein 1
